# Optimal Assessment and Quantification of Iodine Nutrition in Pregnancy and Lactation: Laboratory and Clinical Methods, Controversies and Future Directions

**DOI:** 10.3390/nu11102378

**Published:** 2019-10-05

**Authors:** Creswell J Eastman, Gary Ma, Mu Li

**Affiliations:** 1 Westmead Clinical School, Faculty of Medicine and Health, The University of Sydney, Sydney 2006, Australia; 2 Australian Centre for Control of Iodine Deficiency Disorders (ACCIDD), Faculty of Medicine and Health, The University of Sydney, Sydney 2006, Australia; g.ma@westernsydney.edu.au (G.M.); mu.li@sydney.edu.au (M.L.); 3 School of Medicine (Pathology), Liverpool Hospital Clinical School, Western Sydney University, Liverpool, Sydney 2170, Australia; 4 School of Public Health, Faculty of Medicine and Health, The University of Sydney, Sydney 2006, Australia

**Keywords:** iodine deficiency, thyroid, brain development, pregnancy and lactation, iodine supplementation

## Abstract

Iodine intake must be boosted during pregnancy to meet the demands for increased production and placental transfer of thyroid hormone essential for optimal foetal development. Failure to meet this challenge results in irreversible brain damage, manifested in severity from neurological cretinism to minor or subtle deficits of intelligence and behavioural disorders. Attention is now being focused on explaining observational studies of an association between insufficient iodine intake during pregnancy and mild degrees of intellectual impairment in the offspring and confirming a cause and effect relationship with impaired maternal thyroid function. The current qualitative categorisation of iodine deficiency into mild, moderate and severe by the measurement of the median urinary iodine concentration (MUIC) in a population of school-age children, as a proxy measure of dietary iodine intake, is inappropriate for defining the degree or severity of gestational iodine deficiency and needs to be replaced. This review examines progress in analytical techniques for the measurement of urinary iodine concentration and the application of this technology to epidemiological studies of iodine deficiency with a focus on gestational iodine deficiency. We recommend that more precise definitions and measurements of gestational iodine deficiency, beyond a spot UIC, need to be developed. We review the evidence for hypothyroxinaemia as the cause of intrauterine foetal brain damage in gestational iodine deficiency and discuss the many unanswered questions, from which we propose that further clinical studies need to be designed to address the pathogenesis of neurodevelopmental impairments in the foetus and infant. Agreement on the testing instruments and standardization of processes and procedures for Intelligence Quotient (IQ) and psychomotor tests needs to be reached by investigators, so that valid comparisons can be made among studies of gestational iodine deficiency and neurocognitive outcomes. Finally, the timing, safety and the efficacy of prophylactic iodine supplementation for pregnant and lactating women needs to be established and confirmation that excess intake of iodine during pregnancy is to be avoided.

## 1. Introduction

The trace element iodine is an essential dietary micronutrient for the synthesis of thyroid hormones. Hence, a consequence of sustained deficiency in dietary iodine intake is a reduction in thyroid hormone production and action. Adverse outcomes in humans have been termed Iodine Deficiency Disorders (IDDs), comprising multiple physical, neurological and intellectual deficits with the clinical expression dependent upon the severity, duration and timing of the dietary iodine deficiency at specific life-stages, especially occurring during foetal and infant development [1,2]. While this is true for severe, nutritional iodine deficiency, the effects of mild to moderate iodine deficiency during pregnancy on neurodevelopmental outcomes of the offspring continue to be debated and have recently become a focus of increased interest and research [3]. It should also be emphasised that moderate to severe gestational iodine deficiency increases the risk of spontaneous abortion, low birth weight babies and overall infant morbidity and mortality rates, but the effects of mild to moderate gestational deficiency are less clear [4]. With the widespread acceptance and implementation of prophylactic universal salt iodisation, severe iodine deficiency has been eradicated from most of the developing world, causing a paradigm shift in the epidemiology of IDDs from endemic goitre and cretinism, so well-studied in regions of severe iodine deficiency, to focus on the effects of less severe iodine deficiency, particularly in the developed world [5].

Mild to moderate gestational iodine deficiency has been associated with impaired neurocognitive development, characterised by a decreased Intelligence Quotient (IQ) in the offspring [6,7,8,9,10], but these findings have not been universal [11]. There are multiple possible causes for this lack of certainty, not least being the definitions applied to classification of the severity of maternal iodine deficiency, particularly when it is characterised by an estimation of UIC determined sporadically during gestation coupled with our inability to accurately determine the degree of the micronutrient deficiency by the measurement of other currently used biomarkers [3].

The underlying mechanism causing neurodevelopmental defects is generally believed to be a direct consequence of a lack of thyroid hormone acting on responsive neural tissues during critical stages of foetal development [12]. In turn, the provision of adequate quantities of thyroid hormone for foetal development depends on maternal transfer of the hormone, at least during the first two trimesters of pregnancy until the foetal thyroid gland functions, and this process is directly dependent on an adequate maternal iodine intake throughout the pregnancy [12]. Research has been further hampered by lack of knowledge of thyroid hormone transport across the placenta, coupled with an inability to measure foetal thyroid hormone levels or other biomarkers of thyroid hormone action in the foetus. Thus, much of what is written about mechanisms in humans is inferential or extrapolated from animal studies and requires more convincing evidence in humans.

Iodine supplementation during pregnancy and lactation is widely and justifiably advocated to prevent the potentially devastating and irreversible consequences of iodine deficiency on child development. However, there is some evidence that even mildly excessive intake of iodine during pregnancy in women, who are not severely iodine deficient, may paradoxically cause neurodevelopmental disorders in the offspring [8,13].

The aim of this review is to examine the laboratory and clinical methods used in the assessment and quantification of iodine nutrition during pregnancy and lactation and correlate these instruments with published outcomes that have attributed neurodevelopmental and certain behavioural disorders to iodine deficiency during pregnancy, lactation and infancy.

## 2. Methods for Measuring and Assessing Iodine Nutrition

### 2.1. Measurement of Urine Iodine Concentration (UIC) and Quality Assurance (QA)

There are several analytical methods available for measuring urinary iodine concentration (UIC), most involving an initial step of acid digestion of urine at a high temperature (90–110 °C) to remove any potential interfering substances prior to the spectrophotometric measurement of the Sandell–Kolthoff reactions [14,15]. Earlier methods required chloric acid digestion, generating corrosive and hazardous fumes. Pino et al. recommended using the less hazardous ammonium persulphate as the oxidising agent with subsequent good correlation shown between the two methods [16]. This modified method has been recommended by the World Health Organization (WHO) the United Nations Childrens Fund (UNICEF) and the International Council for Control of Iodine Deficiency Disorders (ICCIDD) for epidemiological assessment of a population and it is the most popular method of choice today [17]. In short, iodine derived from ammonium persulphate digestion at a high temperature is the catalyst that reduces the yellow ceric tetra-ammonium sulphate to a colourless cerous (IV) compound by arsenite, with the rate of disappearance of the ceric ammonium sulphate being inversely proportional to its iodine concentration. This procedure can be carried out either manually or semi automatically or by micro-titre plate format due to the minute quantity of urine sample used in the analysis [15,18]. One of the advantages of using the micro-titre plate protocol for UIC measurement is the overall reduction of specimen volume and chemical usage together with less hazardous chemicals being discharged into the environment.

With increasing concerns about toxic and hazardous chemicals contaminating the environment, efforts have turned to safer, alternative methods for measuring UIC. The application of Inductively Coupled Plasma Mass Spectrometry (ICP&MS) is one such alternative for investigation and diagnosis of diseases in the developed world [19]. This high cost and high maintenance hardware technology is considered the ‘gold standard’ for the measurement of UIC because; (a) it has a high degree of accuracy; (b) it does not require an acid digestion procedure; (c) it is relatively environmentally friendly and; (d) there are no toxic chemicals involved in the ICP&MS method compared to the Sandell–Kolthoff reactions. Urine samples are introduced into a high temperature, inductively coupled plasma source proceeding through various internal processing stages before the aerosol sample is converted into a gaseous phase and then ionised. Any potential interfering substances are therefore eliminated through this procedure [20].

To ensure the sustainability of IDD control programs, it is essential to collect accurate UIC data in population samples and measure iodine content of iodised salt. These require rigorous and sustained laboratory testing procedures and methods. Consequently, a testing laboratory must demonstrate that its results are reliable and reproducible. For practical purposes, each laboratory must establish its own internal quality controls and register for an external assay QA program to monitor assay quality. Intra-assay and inter-assay performance precision should be established to ensure limits are set for day to day variations in terms of incubation time/ temperature and different operator’s behaviour. Once these basic criteria are established, the Levey–Jennings charts should be established to monitor long-term assay precision, result accuracy and assay drift and to take immediate corrective action where required. Ensuring the Quality of Urinary Iodine Procedures Program (EQUIP) is an external quality assurance program that monitors the precision and accuracy of the urinary iodine analyses of participating members. Membership is free of charge and currently it has over 200 life memberships since its inception in 2001. Registration of a laboratory is through the Division of Laboratory Sciences, National Center for Environment Disease Control and Prevention (CDC) in Atlanta, USA [21].

### 2.2. Urine Iodine Concentration (UIC) and Total 24 h Urine Iodine Excretion (UIE) as Proxy Measures of Iodine Intake

Iodine deficiency occurs when iodine intake falls below recommended requirements and is defined for a given population by reference to the median urinary iodine concentration (MUIC) in school-age children in that population [17]. While intake of iodine in food can be estimated indirectly by dietary analysis, the standard method for defining iodine intake is by extrapolation from the measurement of the UIC in a spot urine sample and applying the formula from the Institute of Medicine (IOM) of UIC × 0.235 × body weight or alternatively using the formula UIC × 0.92 × 1.5 that is based upon spot UIC value, average kidney excretion of daily iodine intake of 92% and average daily urine volume of 1.5 L [22]. Measuring a spot UIC, which may vary considerably from day to day, depending on variations in dietary intake and urine volume, and applying these formulae to obtain the total urine iodine excretion (UIE) as a proxy measure of habitual iodine intake only provides imprecise estimates of iodine intake. These facts are frequently overlooked or ignored in scientific publications reporting population iodine status [3]. We have previously expressed the view that reliance on these methods to estimate iodine intake from UIC questions the validity of many published reports that compare populations of different age, gender, ethnic and environmental backgrounds and where these multiple potential confounding variables have been ignored [3]. This is the reason a spot UIC is not acceptable as a diagnostic tool to evaluate an individual’s iodine nutritional status as dietary intake may vary considerably from day to day.

Andersen et al. [23] and Konig et al. [24] demonstrated that to assess individual iodine status more accurately, repeat collections of 10 to 12 separate urine samples over a given time period are needed to give a 20% precision of the result. While this approach is more diagnostically accurate than a single spot UIC, the analysis of many repeat urine samples from an individual is considered a major drawback and limitation with this approach. It has been suggested that the measurement of iodine in a 24 h urine collection for population studies is a more reliable and reproducible method for evaluating individual iodine status than spot UIC [25]. It is also regarded as a better method because urinary iodine excretion (UIE), determined from a 24 h pooled sample, reflects the true daily excretion of an individual. However, in large national iodine nutrition studies there are several drawbacks, including transport of large volumes of samples from site to site make transport logistics difficult to manage. In addition, a high percentage of ‘missed’ samples (incomplete 24 collections) due to the inconvenience of collecting multiple samples in a day. Storage of large and bulky samples in a laboratory is also a concern. The 24 h urine sample collection method does not always reduce intra-individual variability [26,27,28].

Expressing UIC with creatinine as UIC µg/g creatinine or creatinine per day (UIC µg/day creatinine) has been suggested as a better way to express an individual’s iodine excretion level. Creatinine is endogenously produced as a breakdown product of creatine and normally appears in urine at a relatively constant rate over a 24 h period when fluid consumption is regular. Based on this assumption, expressing UIC µg/g creatinine will eliminate any fluctuation of fluid intake. There are other concerns of expressing UIC results with creatinine especially where daily protein intake is low. Indeed, the WHO has stated that the use of creatinine as a correction factor for iodine intake is ‘unnecessary’ and ‘unreliable’ [17]. Creatinine excretion varies with gender, body mass index, ethnic background and most importantly protein intake. Expressing UIC as µg/g creatinine, or µg/day creatinine, may have more merit in pregnancy according to the findings of a study in China where they found that UI/Cr better reflected the 24 h urine iodine excretion and circulating iodine levels in pregnancy rather than the UIC [29].

### 2.3. Role of Neonatal TSH in Assessment and Monitoring of Mild to Moderate Iodine Deficiency

Neonatal thyroid stimulating hormone (nTSH) concentration has been used for approximately five decades for detecting congenital hypothyroidism (CH) [30] and screening programs for CH are almost universal throughout the world. The nTSH concentration was also proposed as a sensitive marker for monitoring iodine deficiency [31]. The WHO/UNICEF/ICCIDD have recommended nTSH, along with UIC, thyroid size and thyroglobulin (Tg), as indicators for detecting and monitoring iodine deficiency in a population [17]. The proportion of nTSH concentration in a newborn population greater than 5 mIU/L above 3% is the cut-off for iodine deficiency. The severity, (mild, moderate and severe) is further defined by the proportion of neonates with TSH concentrations greater than 5 mIU/L. There has been a large body of literature reporting iodine deficiency status or outcomes of iodine prophylaxis programs using nTSH results, with reference to the WHO criteria [32], and in more recent literature [33,34,35,36,37,38]. Most published reports have employed dried capillary blood spots from heel-prick as recommended.

Although this indicator has been widely used for several decades to assess a population’s iodine nutrition status and accepted at its face value, many factors can influence the results. These include maternal smoking and lower weight gain during pregnancy [39], mode of delivery [32], birth weight of the child [33,40], days after birth of sample collection [32,33,34,41], assay methodologies [32] and seasonal variations [33,37]. Further, several studies suggest the proposed cut-off for iodine deficiency should be re-evaluated. For instance, in some countries or areas where pregnant women were known to be iodine deficient, the proportion of nTSH above 5mIU/L was in fact very low [33]. The role of measuring nTSH concentrations for assessing iodine deficiency, monitoring iodine supplementation programs and detecting relevant mild gestational iodine deficiency, is a useful tool, but the assay system must be optimized for these functions, as well as for its primary role in diagnosing CH, and the many confounding variables need to be considered in evaluating the results of nTSH monitoring [32]. Unless these issues are managed correctly, there are always doubts about conclusions reached from retrospective analyses of nTSH data for monitoring of iodine nutritional status of a population.

## 3. Definition and Justification of Mild, Moderate and Severe Iodine Deficiency Status

### Classification of Iodine Deficiency

The epidemiological criteria for classification of the severity of iodine deficiency were initially derived from endemic goitre surveys in representative populations of school-age children and later from correlating total goitre rates with UIC results to derive definitions of iodine deficiency based upon UIC. Total goitre rates of 5.0–19.9% were said to represent mild iodine deficiency, 20.0–29.9% representing moderate iodine deficiency and > 30% representing severe iodine deficiency [17]. We have previously noted that the evidence underpinning these definitions is weak [3]. Laurberg and colleagues in a comprehensive examination of the evidence underlying these assumptions concluded that a cut-off point for urinary iodine excretion (UIE) causing endemic goitre is roughly equivalent to a UIC of 40 µg/L [42] and this not consistent with agreed definitions from international agencies [3]. Multiple confounding factors may be present and provide reasons for the disparity between the presence and severity of endemic goitre and the level of UIC and UIE [42].

Iodine deficiency is now classified by the UIC with the intention of reflecting dietary iodine intake. The recommended dietary intake (RDI) is the estimated amount of iodine required to ensure optimal thyroid function in healthy individuals in specified life stages. The RDI for a healthy adult is 150 µg that corresponds approximately with a spot UIC of 100 µg/L, but this changes in pregnancy. The dramatic and sustained increase in maternal thyroid hormone synthesis and secretion early in the first half of gestation is a response to increased thyroidal stimulation by human chorionic gonadotrophin (hCG) and the necessity to maintain maternal biologically active—“free Thyroxine (FT4)”—concentrations in the face of raised circulating thyroxine-binding globulin (TBG) levels [43]. The MUIC for adequate iodine intake in a pregnant population is 150–249 µg/L, as shown in Table 1. It is interesting that we continue to use the qualitative terms of mild, moderate and severe to describe gestational iodine deficiency, but quantitatively we only have the MUIC figure of 150 µg/L to discriminate between adequate and insufficient iodine intake.

## 4. Mild to Moderate Gestational Iodine Deficiency and Impaired Neurodevelopment in the Offspring

### 4.1. Neurodevelopmental Impairment in Children Resulting from Gestational Mild to Moderate Maternal Iodine Deficiency

Severe iodine deficiency in pregnancy and in childhood has devastating effects on intellectual function in the offspring [44]. Studies conducted in China several decades ago demonstrated that people born and continuing to live in regions of iodine deficiency displayed impaired intellectual and neuromotor development across a broad spectrum of severity [44,45]. In addition to decreased Intelligence Quotients (IQs)—measured by Hiskey Nebraska tests and Griffiths Mental Development Scales—many apparently normal people born in these regions suffered evidence of nerve conduction deafness on audiometry testing and subtle, but detectable, motor disorders demonstrated by careful neurological examination [44,45]. The difference in IQ measurements between iodine-deficient and iodine-replete populations varies considerably in studies conducted in many diverse regions of the world and has been summarised elsewhere [2]. In a meta-analysis of studies conducted in China, measuring the adverse effects of iodine deficiency from populations in many parts of the country, it was reported that intellectual damage in children exposed to severe iodine deficiency was profound, averaging 12.45 IQ points loss [46].

On this background, it is not unexpected that decreased IQ scores, or a “shift to the left” in intelligence testing scores, would be part of a spectrum of neurodevelopmental disorders in regions of gestational iodine deficiency. Accordingly, women who suffer with less severe degrees of iodine deficiency during pregnancy would be more likely to give birth to children with varying, but usually subtle or mild degrees of neurocognitive impairment, such as learning difficulties and decreased IQ scores. Two observational studies undertaken of children in Australia and in the UK have shown an association between mild maternal iodine deficiency and impaired cognition in the offspring [6,7]. In the Australian study, children born to mothers with a UIC of < 150 µg/L had significantly lower educational test scores compared with children born to mothers with UIC > 150 µg/L [6]. Despite growing up in an iodine-sufficient environment (mandatory iodisation of salt used in baking of bread was introduced in Australia in 2009), and experiencing 10 years of schooling, the reduced spelling, grammar and reading outcomes in the affected children persisted into adolescence, confirming the validity of the initial study and the irreversible nature of the neurological damage suffered during gestation [47]. In a similar study conducted in a region of mild iodine deficiency in the UK, similar outcomes were identified in the offspring of mothers whose urinary iodine to creatinine ratio was <150 µg/g. [7]. Both the Australian and British studies provide persuasive evidence of adverse effects of mild maternal iodine deficiency on neurocognitive development in the offspring, but these studies lack data on impaired maternal thyroid function to support a cause and effect relationship [3]. In a large Norwegian based mother and child cohort study, a relationship was found between reduced maternal iodine intake (calculated from food-frequency questionnaires) and reduced motor skills, child language delays and behavioural problems in the offspring [8]. A recent meta-analysis examining the association of maternal iodine status and child IQ from individual participant data of three European prospective birth cohort studies—Generation R (The Netherlands), Infancia y Medio Ambiente Project INMA (Spain), and the Avon Longitudinal Study of Parents and Children ALSPAC (United Kingdom)—found that a lower maternal UI/Cr up to 14 gestational weeks was associated with lower verbal IQ scores in their offspring, assessed between 1.5 and 8.6 years of age [48]. Thus, the data from these observational studies is very persuasive if not definitive. Velasco and colleagues have summarised this very well in explaining why results may vary from one study to another due to the many confounding variables in these studies [49].

As well as impaired IQ in children resulting from mild gestational iodine deficiency, it is reasonable to expect that affected children may also suffer from significant, but less obvious impairments in behaviour, motor function and hearing, as these types of disabilities are characteristic findings in those suffering from neurological cretinism consequent upon severe iodine deficiency in pregnancy, as demonstrated in the studies cited above [44,45]. Unfortunately, a more comprehensive view of subtle forms of brain damage has generally been overlooked and investigations into these potential disabilities requires more attention.

### 4.2. Mechanism of Neurological Damage in Mild to Moderate Gestational Iodine Deficiency

**Figure 1:**
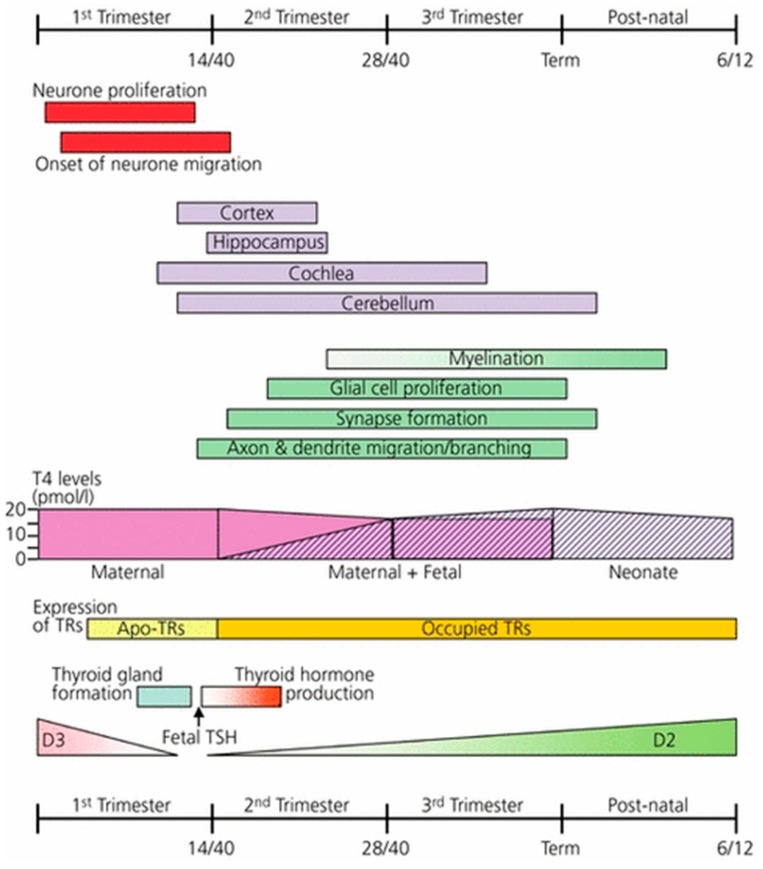
Thyroid hormone action and the development of the brain of the foetus and infant. Relationship between thyroid hormone action and the development of the brain. In the first trimester of pregnancy early neuronal proliferation and migration is dependent on maternal thyroxine (T4). In foetal tissues, inactivating type 3 deiodinase (D3) enzyme expression falls and the development of the thyroid gland commences. By the end of the first trimester, the development of the hypothalamic–pituitary axis has occurred and a surge in thyroid-stimulating hormone (TSH) secretion results in the onset of foetal thyroid hormone production, expression of the activating type 2 iodothyronine deiodinase enzyme (D2) and increasing occupation of thyroid hormone receptors (TRs) by 3,5,3 ¢-L-triiodothyronine (T3). Continuing the development of the brain in the second and third trimesters relies increasingly on T4 produced by both the foetus and mother. Continued post-natal development is entirely dependent on neonatal thyroid hormone production. Apo-TR, unliganded unoccupied thyroid hormone receptor. Reproduced with permission: Williams G.R., Journal of Neuroendocrinology 2008, Vol 20, 784–794, Wiley Publications.

Extensive animal experimental evidence confirms the role of thyroid hormones in brain development [49,50]. The neurodevelopmental and neurophysiological actions of thyroid hormone have been reviewed in detail by Williams [12] (Figure 1). There is good evidence that the major cause of intellectual impairment in moderate to severe iodine deficient environments is from intrauterine brain damage because of decreased thyroid hormone synthesis and action (hypothyroxinaemia). Initially this is due to iodine deficiency in the mother and later in gestation in both the mother and foetus, continuing postnatally in the infant [45,51]. In severe iodine deficiency the expression of the nature and extent of the neurological deficits in humans have been well described [45,52,53]. Except for intellectual impairment, few if any of these neurodevelopmental or other somatic disorders have been reported in mild to moderate iodine deficient populations, possibly because they may often be so subtle, they have not been noted or appropriate examinations and investigations have not been undertaken to detect them. What has been frequently overlooked in clinical research into neurological disorders associated with iodine deficiency is that there are three stages of thyroid hormone dependent neurological development in the human foetus [12], and this issue is particularly relevant to the timing of the initiation of iodine supplementation trials that commence too late in gestation. The first stage of thyroid hormone dependent brain development is from conception to approximately 20 weeks gestation, comprising neuronal proliferation and migration of neurones in the cerebral cortex, hippocampus and medial ganglionic eminence and these processes are dependent upon the action of T4 transferred from the mother presumably very early in gestation. The second stage of neurodevelopment occurs during the second half of pregnancy, and includes neurogenesis, neuronal migration, and multiple other processes of neurological development and differentiation that is dependent upon both maternal and foetal T4 production and action (Figure 1). The third stage of development and brain maturation occurs in the neonatal period and during infancy, and these are entirely dependent on the infant’s T4 production regulated by iodine ingested from breast milk or other external food sources.

The evidence for measurable maternal hypothyroxinaemia, and hence decreased T4 transfer to the foetus in mild iodine deficiency, compared with severe gestational iodine deficiency, has not been consistent and has been lacking in many psychomotor studies designed to assess intellectual impairment in the offspring of mild to moderate iodine deficient mothers. The proposition put forward to explain this is that even a mild degree of iodine deficiency in the mother will result in preferential triiodothyronine (T3) secretion over thyroxine (T4) secretion to conserve iodine, so maintaining euthyroid status in the mother, but resulting in hypothyroidism and brain damage in the foetus that is dependent on T4 and not T3 for normal brain development [49,50]. While this could be the mechanism, there has been limited biochemical evidence in the literature to support this hypothesis. However, in a recent meta-analysis of a cooperative multicentre study determining the effects of thyroid function in early pregnancy on child IQ and autistic traits in a large number of mother pairs from different countries, the investigators found that lower Free T4 levels in the mothers were consistently associated with a lower IQ across the cohorts [54]. Further research is required to establish if maternal levels of thyroid hormones directly correlate with thyroid hormone transfer to foetal brain? The possibility of a non-thyroidal or a thyroid-independent mechanism, such as iodine deficiency itself causing brain damage, has no basis in the published literature. But it is possible that besides iodine other micronutrient deficiencies, such as iron and selenium for example, may play a small part in the development of thyroid hypofunction and the pathogenesis of neurodevelopmental abnormalities [55].

### 4.3. Controversies and Unanswered Questions

#### Isolated Hypothyroxinaemia

Pop and co-workers were the first to show that children born to hypothyroxinaemic women (Free T4 levels below the 10th percentile, with TSH levels within the normal reference range, during the first trimester of pregnancy) are at risk of having both mental and motor development delay when tested at 1 year of age [56]. The reason that serum TSH concentrations were not elevated, as would be expected with subnormal circulating Free T4 levels, was not clear nor was the possible cause of the isolated hypothyroxinaemia ever determined. It is difficult to understand why the maternal TSH level is not elevated in response to a decreased Free T4 level, even if there is a minor preferential increase in thyroidal T3 secretion. Anecdotally, isolated hypothyroxinaemia is frequently said to be a result of maternal iodine deficiency, but there is little clinical research evidence to support this belief. Because of well-documented methodological problems encountered in the measurement of Free T4 concentrations in pregnancy, international clinical guidelines do not routinely recommend this test in pregnant women [57,58]. The certainty of isolated hypothyroxinaemia as a specific pathological entity remains in question as the ATA guidelines do not recommend thyroxine replacement therapy for this condition [59].

### 4.4. Attention Deficit Hyperactivity Disorders (ADHDs), Autism and Other Behavioural Disorders

There have been several reports in developed countries linking attention deficit hyperactivity disorders in the offspring of mothers exposed to mild to moderate iodine deficiency during, pregnancy suggesting a possible cause and effect relationship between these entities [48,60]. Suggested mechanisms include reduced intracellular brain T3 from inadequate transfer of maternal T4 or alternatively reduced sensitivity of foetal brain nuclear receptors to T3. In the Generation R population-based birth cohort study in the Netherlands, designed to identify early environmental and genetic determinants of growth and development from foetal life onwards, there was 4-fold increase in the risk of developing autistic behaviour in children born to hypothyroxinaemic mothers [54]. However, the reverse effect has been reported in the large Norwegian Mother and Child Cohort Study where the increased risk of ADHD was seen in children born to mothers who had commenced taking an iodine supplement during pregnancy [61]. The authors postulated that the mechanism of this effect could be explained by a temporary lower thyroid hormone production due to an acute higher iodine availability during the vulnerable period of neurodevelopment, suggesting a possible adverse effect of exposure to iodine excess [62]. Increased rates of ADHD and autism were not reported in the Australian and UK studies that reported decreased IQ measurements in the children of mildly iodine deficient mothers [6,7].

## 5. Iodine Supplementation during Pregnancy and Lactation: Why, How Much and When?

In the first trimester the foetus is completely dependent upon maternal T4 transferred across the placenta for its thyroid hormone needs. By mid-gestation the foetal thyroid is capable of concentrating iodine derived from the mother and synthesising limited quantities of thyroid hormones with a gradual increase in production continuing until birth. All the evidence suggests that iodine supplementation is most effective—and possibly only effective—in preventing foetal neurological damage if it is commenced before conception or in the first trimester and early in the second trimester of pregnancy and continued throughout gestation [48,53].

The assumption underlying the recommendations for increasing iodine intake during pregnancy is that the estimated 50% increase in maternal thyroid hormone production requires a commensurate increase in iodine intake from the daily pre-pregnant RDI of 150 µg to 225 µg, rounded out to 250 µg, by many expert guidelines [57,58]. Most published clinical guidelines recommend a daily oral iodine supplement of 150 µg, taken in the form of potassium iodide or potassium iodate, for women who live in countries without an established universal salt iodisation program [57,58]. In turn, a UIC of 150 µg/L would theoretically represent an RDI of approximately 250 µg. This reference UIC value for pregnancy is not based on any direct experimental evidence and the advice for a supplement of 150 µg is simply a best estimate “to close the gap” between a deficient daily intake and the optimal intake for pregnancy. While there is a paucity of scientific studies to support this recommendation, our studies in Australia where modelling iodine intakes, based on both dietary and biochemical UIC data, showed that a daily iodine supplement of 100 to 150 µg for Australian women would achieve the optimal daily target intake of 250 µg for pregnant Australian women [63]. Of course, this recommendation refers to pregnant women in Australia and may not apply to different populations or subgroups in other populations where pre-pregnancy iodine intakes may vary considerably.

The evidence for any direct benefit of iodine supplementation on physical and biochemical outcomes in women with mild to moderate gestational iodine deficiency has been shown in randomised trials, where daily administration of 100 µg potassium iodide prevented the development of maternal goitre and prevented a rise in serum thyroglobulin (Tg) and TSH levels [64]. By contrast, with severe iodine deficiency, evidence for beneficial outcomes in mild to moderate iodine deficiency of prenatal or periconceptual iodine supplementation on growth and improved neurocognitive development in the offspring remains lacking [65]. Indeed, in a Spanish study, infant neuropsychological development tested at one year of age was not improved in children whose mothers were given iodine supplements during pregnancy [11]. Concerns about conclusions from this study relate to the use of the Bayley Scales of Infant Development at this early age for predicting later cognitive performance. It has been emphasised that while contemporary RCTs of iodine supplementation in pregnancy measuring outcomes addressing childhood development are indicated, conduct of such RCTs may not be feasible in populations where iodine supplementation in pregnancy is widely practiced [65]. There are also ethical and practical issues of conducting an RCT in pregnant women when iodine supplementation in pregnancy has been universally promoted by medical and scientific bodies. The much awaited results of a randomised, double-blind, placebo-controlled trial of the effects of iodine supplementation conducted in India and Thailand have recently been published [66]. The iodine supplement was 200 µg daily from about 10 weeks gestation until delivery. It is noteworthy that the median MUIC for the women entered into the study was 131 µg/L at baseline. The median UIC in the Thai women (*n* = 514) was in the deficient range at 112 µg/L, but in the Indian women (*n* = 318) the MUIC was in the iodine-sufficient range at 188 µg/L, and these women probably should have been excluded from the study. The data suggests that it is likely that the women in the placebo arm of the study also had an increased intake of iodine during their pregnancies, as iodine replacement programs in both countries likely also confounded the outcomes. It is disappointing that this trial has not provided definitive answers to the questions being asked, but further confuses the issue. In a Commentary on the trial written in the Lancet, it was stated “that the trial cannot be considered as conclusive evidence that iodine supplementation has no benefit in mild to moderately iodine deficient pregnant women in terms of offspring neurodevelopment” [67].

The timing of iodine supplementation in relation to gestation appears to be critical. Given the timing of thyroid hormone action on the developing foetal brain (Figure 1), it makes sense that iodine supplementation to potentially iodine deficient women should commence, where possible, well in advance of conception to ensure adequate thyroidal iodine stores are available to withstand short periods of deficient iodine intake during pregnancy, particularly during the first trimester, when “morning sickness” may interfere with iodine intake. We have previously emphasised that total thyroidal iodine store is the more important parameter in the maintenance of optimal thyroid hormone secretion during pregnancy rather than the day-to-day dietary intake [3].

### 5.1. Is Iodine Supplementation Safe during Pregnancy?

The recommended upper safe level of intake during pregnancy is 1100 µg per day. It is the same as the non-pregnant state, but this quantity is likely to be an excessive intake for pregnancy. A WHO expert consultation group arbitrarily recommended a safe upper limit of 500 µg iodine daily during pregnancy, but this appears to be a result of weight of opinion, rather than scientific evidence, as we can find no convincing studies defining safe upper limits of iodine intake during pregnancy and lactation [23]. However, a recent large cross-sectional study from China demonstrated in pregnancy the well-known, U-shaped relationship between thyroid function and UIC with thyroid function being optimal in their study group when UIC was between 150 and 250 µg/L [68]. From this study they concluded that the upper limit of UIC should not exceed 250 µg/L, as women with a UIC > 250 µg/L had a significantly increased risk of developing subclinical hypothyroidism and women with a UIC > 500 µg/L had an increased risk of isolated hypothyroxinaemia. A recent study of pregnant women in Brazil showed women with excessive iodine intake during pregnancy had an increased risk of developing subclinical hypothyroidism, specifically those with a UIC > 500 µg/L [13]. The published data supports the WHO recommendations to avoid an excessive iodine intake in pregnancy.

In general, an iodine intake in excess of normal daily requirements is well tolerated by a healthy individual free of any underlying thyroid disorder, as occurs commonly in countries such as Japan and Korea where habitual iodine intake is high. Transient inhibition of thyroid hormone production and release in response to exposure to excessive iodine is called the acute Wolff–Chaikoff effect [69]. Typically, the thyroid “escapes” from the acute Wolff–Chaikoff effect within a few days or weeks through downregulation of the iodide transporter in thyroid cells, and then recovers with resumption of normal thyroid hormone synthesis. Persistent thyroidal dysfunction may be precipitated in some individuals with underlying autoimmune or nodular thyroid disease when they are exposed to excessive iodine intake on a continuing basis, as reported after implementation of population iodisation programs in regions of long-standing iodine deficiency [70]. Further, euthyroid goitre may occur in people from lifelong exposure to excessive iodine intake as has been documented from environmental causes such as high iodine content of drinking water [71,72]. Concerns are frequently raised of potential risks to the foetus of iodine supplementation during pregnancy as the foetal thyroid gland is more vulnerable to exposure from excessive quantities of iodine than the normal adult thyroid gland [65]. It is possible that any injurious effect on the foetus may be due to both dosage and duration of iodine administration, but this remains to be adequately investigated. It is noteworthy that in the RCT of iodine supplementation during pregnancy conducted in India and Thailand monitoring of thyroid function in the women receiving a daily iodine supplement of 200 µg did not reveal any disturbances in these parameters [66].

The WHO has recommended large “one off” pharmacological doses of iodine in mg quantities, administered in the form of iodised oil, to prevent iodine deficiency in pregnant women in many parts of the developing world where Universal Salt Iodisation has not been established and where moderate to severe iodine deficiency is prevalent and where daily iodine supplementation is impracticable [23,73]. Concerns have been raised that excessive iodine intake from iodised oil administered early in pregnancy could suppress maternal thyroid hormone secretion through the Wolff–Chaikoff effect and, if administered later in pregnancy, cause a similar effect on the developing foetal thyroid gland at a time when the foetus is becoming dependent on its own thyroid hormone secretion for normal development. From available evidence, the WHO concluded that “iodised oil administration to women before or at any time during gestation has no harmful side effects”. Moreover, it was concluded that iodised oil not only prevents endemic cretinism and mental retardation in infants due to iodine deficiency, but also decreases foetal and perinatal mortality and increases birth weight [23]. Thus, there is conflicting information in the literature regarding potential harm to mother and baby of an iodine intake more than requirements during pregnancy. Irrespective of these recommendations, with iodine supplementation “more is not better” during pregnancy and excessive intake should be avoided where possible.

### 5.2. Iodine Supplementation during Lactation

Adequate production of thyroid hormone in infancy is critical for the continuation of somatic and brain growth and development during infancy [51]. Iodine deficiency in infancy presents a serious threat to neurological development because thyroid hormone requirements, and hence iodine, are greatest per kg body weight at that time than at any other life stage [74]. While ensuring adequate iodine intake during infancy is crucial in preventing the adverse effects of postnatal hypothyroidism, supplementation will not reverse the neurological damage from intrauterine thyroid hormone deprivation [44,53]. The supply of iodine to the infant comes from breast milk that is dependent on the dietary iodine intake of the mother [74]. From an analysis of published work, Delange concluded that the iodine RDI in neonates is 90 µg/day and the median UIC to be expected in the mother when this requirement is met is 180–225 µg/L, a value comparable to the one recommended for pregnant women [23,74].

For lactating women, WHO, UNICEF, and ICCIDD/IGN recommend a daily dose of 250 μg as potassium iodide. Alternatively, in countries where iodine deficiency is evident and not corrected by an established USI program, these authorities recommend one annual depot dose of 400 mg of iodised oil administered orally at 6 monthly intervals for the exclusively breastfeeding mother [23]. The assumption is that with these supplements, breast milk will deliver the recommended daily iodine intake of at least 90 µg to the infant. In a double blind, randomised placebo-controlled trial in a moderate to severe iodine deficiency area in Morocco, Bouhouch and colleagues showed that a 400 mg dose of iodised oil administered orally to breastfeeding women provided optimal amounts of iodine to their infants for 6 months without causing any adverse effects in mothers or infants [75]. In this landmark study indirect iodine supplementation via breast milk proved superior to direct supplementation of the infants.

## 6. Conclusions and Future Directions

### 6.1. Diagnosis and Classification of Iodine Deficiency

The need for a revamped classification system and terminology for IDDs, particularly those disorders occurring because of gestational iodine deficiency, is long overdue [3]. It is readily apparent by doing some simple arithmetic calculations that the application of the definition of gestational iodine deficiency as an MUIC of <150 µg/L can be very misleading in different ethnic populations of varying weights and varying urine volumes. It is unrealistic to rely upon on the result of a single spot UIC sample, collected randomly during gestation, to characterise the iodine nutritional status of a pregnant woman and expect this result to correlate with adverse outcomes in her offspring. Therefore, more precise definitions and measurement systems beyond a spot UIC need to be developed to identify the presence and gradations of severity of gestational iodine deficiency.

### 6.2. Paradigm Shift

Until Hetzel devised the term IDD, the relationship between severe iodine deficiency, goitre and irreversible brain damage, as manifested by neurological endemic cretinism, had long been accepted but was not well understood [1]. The concept of a diverse spectrum of disorders, gathered together as IDDs, brought about a paradigm shift towards understanding and defining the mechanisms and more subtle neurological and behavioural manifestations of brain damage caused by gestational iodine deficiency [51,52,53]. Given the complexity and extent of thyroid hormone action on the developing brain and the multiple severe neurologic abnormalities described in neurological endemic cretinism caused by a deficiency of thyroid hormone, it is not unexpected that there has been a further paradigm shift over the past two decades recognizing that mild and subtle forms of IDDs are not confined to remote, mountainous areas in developing countries, but are global public health problems affecting the developed world [5]. Unfortunately, the definitions and quantification of the neurological and psychological consequences of less severe iodine deficiency during gestation and infancy remain contentious and uncertain. Velasco and colleagues have discussed the many potential problems confounding the interpretation of data from studies on mild or borderline, gestational iodine deficiency [49]. The selection of subjects, differing diagnostic testing instruments for IQ and behavioural disorders, and the inclusion of results from poorly designed and flawed studies have been significant confounding factors. There needs to be agreement on the various testing instruments and standardization of processes and procedures, particularly for IQ and behavioural testing, so that valid comparisons can be made among studies of gestational iodine deficiency outcomes.

### 6.3. Pathogenesis of Neurodevelopmental Disorders in the Foetus and Infant

There is no credible alternative to decreased thyroid hormone production and action as the cause of impaired neurological and physical development in the foetus and infant from nutritional iodine deficiency. Intrauterine brain damage that occurs predominantly in the first half of pregnancy is due to inadequate transfer of thyroid hormone from the mother which is compounded in the second half of pregnancy, presumably due to compromised foetal thyroid hormone production. Postnatal impaired neurological and physical development in the infant is a direct consequence of inadequate thyroid hormone production by the infant’s thyroid [51]. This is the basis for the focus from the time of conception, or even before conception, through infancy to prevent irreversible brain damage from iodine deficiency. While this research has been conducted in severely iodine populations in developing countries, convincing biochemical evidence from comprehensive, longitudinal studies is lacking in mild iodine deficiency. If mild gestational iodine deficiency causes significant neurodevelopmental impairment in the foetus resulting from maternal hypothyroxinaemia, it would be expected that these women would suffer from overt or subclinical hypothyroidism, but there is little biochemical evidence to support this proposition. The body’s compensatory response to iodine deficiency and a consequent decrease in circulating free thyroxine levels, even though this decrease may be minor, is to increase pituitary TSH secretion and thyroid mass. Why this does not appear to be the case in reports of women with mild gestational iodine deficiency giving birth to children with impaired neurocognitive function remains unanswered. Future clinical research studies need to be designed to address this issue.

### 6.4. Iodine Supplementation to Prevent Gestational Iodine Deficiency Disorders

The timing of the administration of an appropriate iodine supplement early in pregnancy to prevent neurological damage in the offspring was demonstrated many years ago in field studies in Papua New Guinea [1]. Since then, there has been an abundance of observational studies demonstrating the efficacy of iodine supplementation, before conception or early in gestation, in preventing IDDs especially in regions of severe iodine deficiency. Good quality evidence from well conducted trials is lacking in gestational mild to moderate iodine deficiency. A recent Cochrane Review of 11 trials involving more than 2700 women taking iodine supplements during pregnancy in settings of mild to moderate iodine deficiency did not show any differences in birth weight or thyroid function [76]. From a systematic review of randomised controlled trials of iodine supplementation during pregnancy and the periconceptual period, Zhou and colleagues concluded that in regions of severe iodine deficiency there were reductions in the rates of cretinism and some motor functions in the offspring, but no improvements in childhood intelligence, growth and development or pregnancy outcomes and that the effects of iodine supplementation on thyroid function of mothers and their children were inconsistent [65]. In short, there is a lack of high-quality evidence supporting the dosage and the efficacy of iodine supplementation in mild gestational iodine deficiency, possibly because of poorly designed studies and interventions commencing too late in gestation [49]. In our current state of uncertainty, iodine supplements in excess of pregnancy requirements are potentially harmful and should be avoided.

### 6.5. Key Messages

- More precise definitions and measurements of gestational iodine deficiency, beyond a spot UIC, need to be developed.
- Agreement on the testing instruments and standardization of processes and procedures for IQ and psychomotor tests need to be reached in the medical and scientific community, so that valid comparisons can be made among studies of gestational iodine deficiency neurocognitive outcomes.
- Clinical studies need to be designed to address the pathogenesis of neurodevelopmental impairments in the foetus and infant.
- The safety and the efficacy of iodine supplementation in mild gestational iodine deficiency need to be established and excess intake of iodine during pregnancy is to be avoided.

## Figures and Tables

**Table 1:** Median urinary iodine concentration (UIC) categories in non-pregnant, pregnant and lactating women.

Population Group	Median UIC (μg/L)	Category of Iodine Intake
Non-Pregnant women	<100 >100	Insufficient Adequate
Pregnant women	<150 150–249 250–499 ≥500	Insufficient Adequate More than adequate Excessive ^‡^
Lactating women	<100 ≥100	Insufficient Adequate

^‡^ Means excess of the amount required to prevent and control iodine deficiency.
